# Chromatin Composition Is Changed by Poly**(**ADP-ribosyl**)**ation during Chromatin Immunoprecipitation

**DOI:** 10.1371/journal.pone.0032914

**Published:** 2012-03-30

**Authors:** Sascha Beneke, Kirstin Meyer, Anja Holtz, Katharina Hüttner, Alexander Bürkle

**Affiliations:** 1 Molecular Toxicology, University of Konstanz, Konstanz, Germany; 2 BioImaging Center, University of Konstanz, Konstanz, Germany; The University of Hong Kong, Hong Kong

## Abstract

Chromatin-immunoprecipitation (ChIP) employs generally a mild formaldehyde cross-linking step, which is followed by isolation of specific protein-DNA complexes and subsequent PCR testing, to analyze DNA-protein interactions. Poly(ADP-ribosyl)ation, a posttranslational modification involved in diverse cellular functions like repair, replication, transcription, and cell death regulation, is most prominent after DNA damage. Poly(ADP-ribose)polymerase-1 is activated upon binding to DNA strand-breaks and coordinates repair by recruitment or displacement of proteins. Several proteins involved in different nuclear pathways are directly modified or contain poly(ADP-ribose)-interaction motifs. Thus, poly(ADP-ribose) regulates chromatin composition. In immunofluorescence experiments, we noticed artificial polymer-formation after formaldehyde-fixation of undamaged cells. Therefore, we analyzed if the formaldehyde applied during ChIP also induces poly(ADP-ribosyl)ation and its impact on chromatin composition. We observed massive polymer-formation in three different ChIP-protocols tested independent on the cell line. This was due to induction of DNA damage signaling as monitored by γH2AX formation. To abrogate poly(ADP-ribose) synthesis, we inhibited this enzymatic reaction either pharmacologically or by increased formaldehyde concentration. Both approaches changed ChIP-efficiency. Additionally, we detected specific differences in promoter-occupancy of tested transcription factors as well as the in the presence of histone H1 at the respective sites. In summary, we show here that standard ChIP is flawed by artificial formation of poly(ADP-ribose) and suppression of this enzymatic activity improves ChIP-efficiency in general. Also, we detected specific changes in promoter-occupancy dependent on poly(ADP-ribose). By preventing polymer synthesis with the proposed modifications in standard ChIP protocols it is now possible to analyze the natural chromatin-composition.

## Introduction

The method of chromatin immunoprecipitation (ChIP) is widely used to monitor changes in chromatin composition. By mild treatment of cells with formaldehyde, covalent protein-DNA cross-links are formed. After lysis chromatin is fragmented by sonication and antibodies are used to precipitate protein-DNA complexes. Subsequently, DNA is isolated and analyzed by PCR regarding the presence of specific sequences [1]. We detected in formaldehyde-fixed cells the biopolymer poly(ADP-ribose) without the application of genotoxins. In general, this enzymatic product can only be observed in cells directly after treatment with DNA damaging agents, as its abundance in unstressed cells is below detection limit. Poly(ADP-ribosyl)ation (PARylation) is a posttranslational modification of proteins catalyzed by the family of poly(ADP-ribose)polymerases (PARPs) [2], [3] and consists of protein-coupled, linear or branched chains of covalently linked ADP-ribose units synthesized from NAD^+^ [4]. PARylation regulates processes such as transcription [5]–[11], replication [12], vesicle trafficking [13], telomere maintenance [14], [15], mitosis [16]–[19], cell death [20] and chromatin organization [21]–[29], but most prominent is this enzymatic reaction in DNA repair [30]. Binding to DNA single-strand and double-strand breaks as induced by genotoxins or during replication stimulates the enzymatic activity of PARP1 and PARP2. Main acceptors of PARylation are histones and PARPs themselves, but many more proteins have been described as targets. While some acceptor proteins are covalently modified by PAR, a large number of proteins interact with PAR non-covalently [31]–[34], and in either case, protein function is altered. Covalent modification inactivates the acceptor in general, whereas the effect of non-covalently bound PAR can be diverse. For example, the base-excision repair platform protein XRCC1 is attracted by PAR to damaged sites [35], whereas nucleosomes are disassembled due to the high affinity of histones to PAR [36], thus opening up chromatin. Macro-domain containing proteins like the histone variant macroH2A [29] and the chromatin remodeler Alc1 [27] can bind poly(ADP-ribose) in a capping like fashion and accumulate at sites of PAR synthesis. Additional PAR binding motifs are a PAR-binding Zinc-finger (PBZ) [33] and a conserved sequence of basic and hydrophobic amino acids [31]. Next to the regulation of base-excision repair, PAR is necessary for full activation of ATM [37] and recruitment of signal transmission factors [38]. As damage-dependent PAR formation is crucial for single-strand break and base-excision repair, PARP inhibition is applied in tumor therapy [39], [40].

Here we show that formaldehyde commonly used as fixative in ChIP methods induces strand breaks and massive PAR synthesis, altering ChIP results. Changing the protocol by adding PARP inhibitors or using a more stringent fixation regimen prevents this PARylation and alters not only the amount of proteins cross-linked to DNA, but also relative promoter occupancies. Our data provide evidence that standard ChIP procedures are flawed by induction of PAR formation, which changes chromatin composition. Therefore, data obtained with conventional chromatin-immunoprecipitation protocols have to be interpreted with caution.

## Results

**Figure 1:**
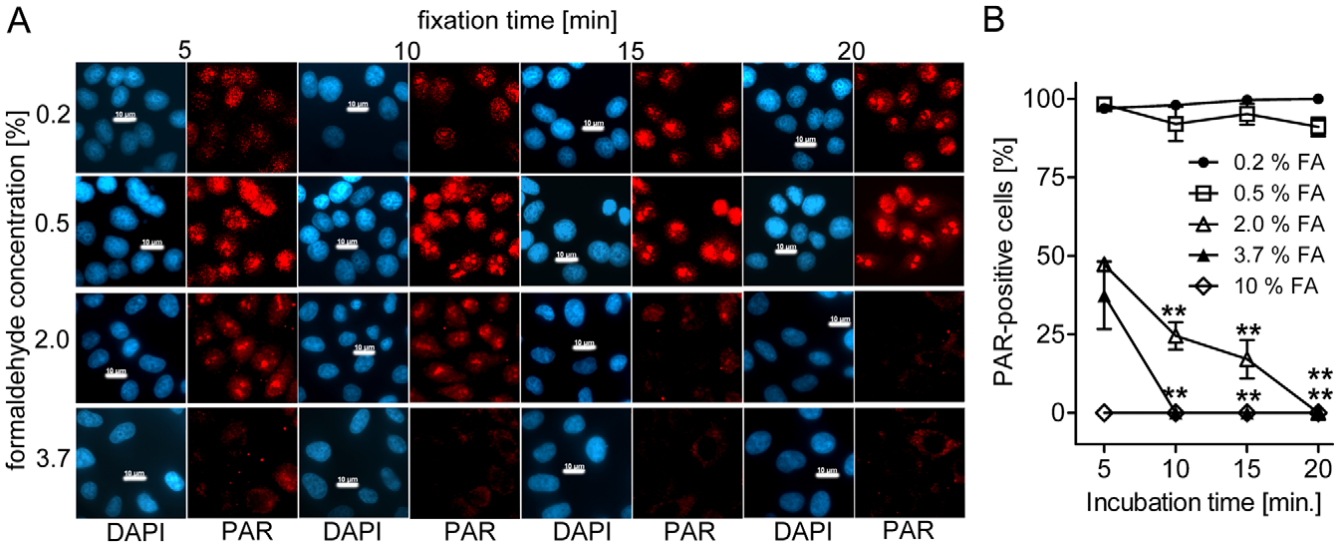
Low formaldehyde concentration and short fixation induce PAR formation. (**A**) HeLaS3 cells were fixed with increasing formaldehyde concentrations and for indicated times. Formaldehyde concentrations are indicated on the left side, fixation time on top. DAPI = nucleus; PAR = poly(ADP-ribose). Scale bars represent 10 µM. (**B**) Evaluation of three independent experiments (N = 3) with at least 50 cells per N as in (A) including 10% formaldehyde (FA) values. Increasing fixation time using 2% FA and 3.7% FA leads to significant decrease in PAR formation. Means were compared to the respective 5 min value. Error bar represent mean±s.e.m., **P<0.01; data were analyzed with one-way ANOVA and Dunnett's Multiple Comparison Test.

The presence of poly(ADP-ribose) in cells is in general below detection limit in immunofluorescence studies without genotoxic treatment. To observe PAR formation after application of DNA-damaging agents, standard fixation protocols include either alcohol (methanol or ethanol) or 10% trichloroacetic acid. If formaldehyde is used as a fixative similar to experiments aiming at protein localization, we often noticed false positive PAR signals in control cells not exposed to genotoxins. In order to analyze this finding more closely, we used different concentrations of formaldehyde ranging from 0.2% to 10% and varying fixation times between 5 to 20 min (Figure 1A). Our data reveal that under these conditions PAR formation in HeLaS3 cells occurs independently of genotoxic treatment and is inversely correlated to formaldehyde concentration and duration of fixation, i.e. high formaldehyde concentrations reduce the time necessary to quench polymer production. 5 min of 2% formaldehyde resulted in only 50% PAR-positive cells, with a decrease down to 0% at 20 min. Using 3.7% formaldehyde, 10 min incubation time was already sufficient to completely suppress PAR formation (Figure 1B).

**Figure 2:**
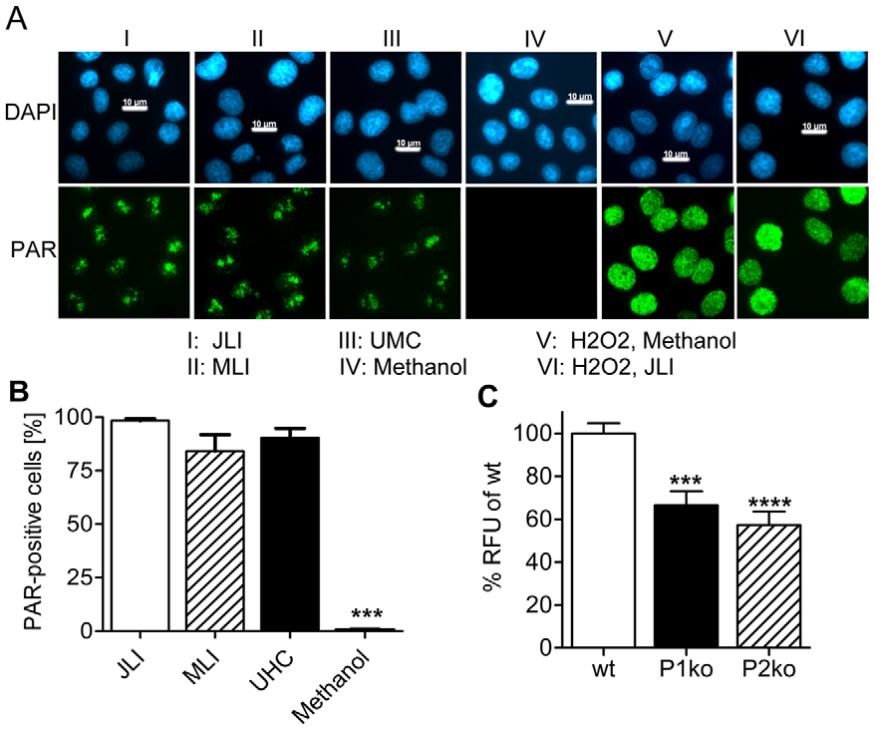
Fixation by ChIP protocols induces PARylation. (**A**) PAR staining after fixation by three different ChIP protocols or methanol. ChIP fixations induce PAR staining (I–III). Methanol fixation shows no PAR formation (IV). H_2_O_2_ and methanol fixation (V) induces granular PAR staining. Focal PAR formation in JLI fixation (I) is reverted to normal distribution if H_2_O_2_ is applied in advance (VI). Procedures are indicated below microscopic pictures. Scale bars represent 10 µM. (**B**) Statistical evaluation of data obtained in (A). Three independent experiments (N = 3) were analyzed with at least 50 cells each data point of one experiment. % PAR positive cells were calculated and analyzed by one-way ANOVA and Bonferroni's Multiple Comparison Test; ***P<0.001. Only methanol fixation is significantly different from the others. Error bars represent mean±s.e.m. (**C**) Mouse 3T3 cells were fixed by JLI protocol. PAR formation was detected in all three lines tested, i.e. wild type (wt), *PARP1* knockout (P1ko) and *PARP2* knockout (P2ko). PAR-fluorescence intensities of cells from four randomly chosen microscopic fields per cell line were analyzed by ImageJ and normalized to intensity in wt cells (RFU: relative fluorescence units). At least six independent experiments were used for statistical analysis by One-Way-ANOVA with Bonferroni's Multiple Comparisons Test; error bars represent mean±s.e.m. (N≥6), ***P<0.001 wt vs. P1ko, ****P<0.0001 wt vs. P2ko, P1ko vs. P2ko not significant.

**Figure 3:**
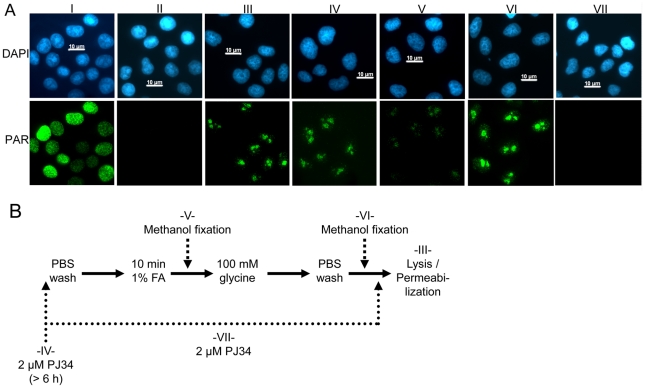
PARP inhibition suppresses PAR formation only if present in every step. (**A**) Detection of PAR by immunofluorescence after different fixation strategies. H_2_O_2_ in combination with methanol fixation induces an even distribution of PAR-staining within the nucleus (I). Pretreatment of cells with 2 µM PJ34 6 h in advance of damage induction and methanol fixation completely suppresses PAR formation (II). Fixation of cells with JLI protocol induces polymer synthesis without H_2_O_2_ (III), but in contrast to (II), PJ34 is not able to block PARP activity completely (IV). Methanol fixation directly after the formaldehyde step reduces PAR staining (V), but not if the cells were fixed after PBS washing (VI). PJ34 is able to suppress PAR formation only if present in all steps until lysis (VII). Scale bars represent 10 µM. (**B**) Flow chart of the different fixation strategies. Standard JLI fixation (III) encompasses all steps until lysis/permeabilization for immunofluorescence detection. Preincubation with 2 µM PARP inhibitor PJ34 (IV) is otherwise identical to (III). Methanol is used to fix cells either directly after formaldehyde treatment (V), or after PBS washes (VI). (VII) 2 µM PJ34 is used for preincubation and continuous treatment of cells during all steps until lysis/permeabilization for immunofluorescence detection.

Having established the link between PARP activity and low-dose formaldehyde fixation, we focused on the technique of chromatin immunoprecipitation (ChIP). In this method, a 10 minute/1% formaldehyde fixation step is typically used to crosslink proteins with DNA. We analyzed three different published ChIP protocols abbreviated JLI, MLI and UMC, respectively [41]–[43] and tested them for induction of PAR formation (Figure 2A). As summarized in Fig. 2B, nearly 100% of the cells were polymer positive in all three protocols, whereas standard methanol fixation showed no signal. To exclude that PAR formation is confined to HeLa cells and to define, which PARP is the prevalent enzyme performing this reaction, we employed mouse 3T3 fibroblasts and subjected them to JLI fixation procedure (Figure 2C). Wild type (wt) cells also showed massive PAR synthesis with formaldehyde, similar to HeLa cells. Genetic deletion of either PARP1 protein (P1ko) or PARP2 protein (P2ko) reduced PAR levels to a similar extent of about 40%, which suggests that at least these two PARPs are responsible for synthesis of polymer after low-dose formaldehyde treatment. In order to abrogate PAR synthesis, we used the standard treatment to suppress polymer production after DNA damage by applying 2 µM PJ34, a pan-PARP inhibitor, 6 h in advance of fixation (Figure 3). Still, we observed polymer signals in all cells after formaldehyde fixation. In order to determine at which point of the JLI protocol [42] PAR is produced, we fixed the cells with methanol directly after the formaldehyde or the PBS washing step, respectively. Only methanol application directly after formaldehyde fixation reduced PAR formation significantly. Therefore, PARP activity is triggered during formaldehyde incubation and aggravated during PBS washings, as PAR intensity is massively increased. This enzymatic reaction could only be blocked by a combined pre- and post-incubation with the inhibitor (Figure 3A, panel VII). This suggests that the fixation process itself induces PARP activity.

**Figure 4:**
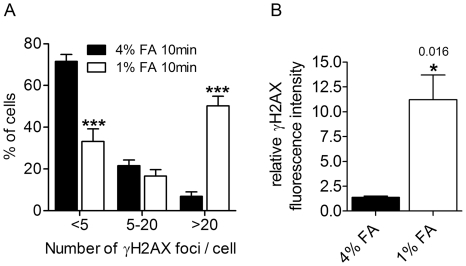
Low-dose formaldehyde induces γH2AX formation. Cells were fixed with 1% or 4% paraformaldehyde and analyzed by confocal microscopy. (**A**) γH2AX foci were counted using one confocal slice after reducing background staining by ImageJ software. Reduction parameters were identical for respective pictures. Cells were split into three groups with (i) less than 5, (ii) between 5 and 20, (iii) more than 20 foci, and percent of total cells was calculated. 10 min 1% paraformaldehyde (1% FA) induces more than sevenfold increase in cells with more than 20 foci, and a decrease in cells with less than 5 foci compared to 4% paraformaldehyde (4% FA). Error bars represent mean±s.e.m. (N = 3), ***P<0.001; data were analyzed with one-way ANOVA and Dunnett's Multiple Comparison Test. (**B**) All pictures from a z-stack were analyzed for γH2AX foci intensity and normalized to cells fixed for 20 min with 4% paraformaldehyde. Intensity increases eightfold with 1% FA compared to 4% FA. Error bars represent mean±s.e.m. (N = 3), *P = 0.016; data were analyzed by unpaired t-test.

In the next step, we investigated if formaldehyde fixation induces DNA damage and related signaling by analyzing γH2AX formation with confocal microscopy. We detected a more than sevenfold increase in cells with high amounts of γH2AX foci if treated with 1% formaldehyde compared to 4% formaldehyde, reflecting massively induced DNA strand-break signaling (Figure 4A). The overall γH2AX intensity in the whole nucleus increased eightfold (Figure 4B). Co-localization analysis in HeLa S3 cells as well as VH7 normal human fibroblasts revealed that both DNA damage markers work independently (Figure S1A–D, see Materials and Methods S1 for experimental setup), with very low if any overlap. Additionally, short term PJ34 application before fixation suppressed synthesis of PAR, but not of γH2AX foci, whereas 3.7% formaldehyde abrogated appearance of both DNA damage markers (Figure S1A–D). Thus, PARP activity seems to be induced by the formation of different DNA lesions than γH2AX, but both signaling processes are abrogated at high concentrations of formaldehyde.

**Figure 5:**
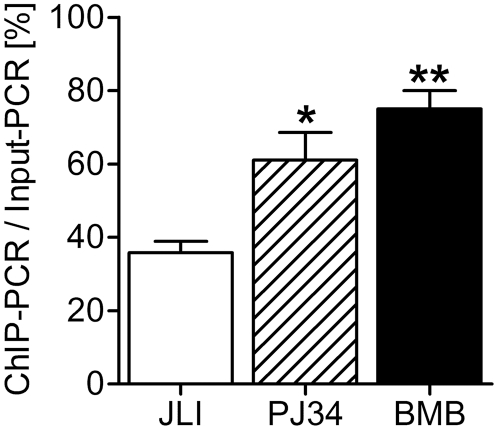
Suppression of PARylation impacts on ChIP efficiency. Evaluation of ChIP efficiency of PARP1 bound to *PARP1* promoter. Both modifications improve significantly ChIP efficiency. Error bars represent mean±s.e.m. (N = 3), *P<0.05, **P<0.01; data were analyzed with one-way ANOVA and Bonferroni's Multiple Comparison Test.

In order to determine the impact of formaldehyde-induced PARP activity on ChIP efficiency, we designed two modified versions of a common ChIP method on the basis of the JLI protocol. We compared the original protocol with one including 2 µM of the PARP inhibitor PJ34 in all steps until lysis (PJ34), starting from 6 h ahead of sample processing as the only variation. Another modification was fixation for 10 min with 3.7% formaldehyde (BMB) instead of 1%. In order to obtain in this case comparable results for chromatin fragmentation, sonication cycles had to be increased two- to fourfold to achieve a similar pattern in DNA fragment length. Otherwise, treatment was identical to standard JLI protocol. To test our hypothesis that formaldehyde fixation alters ChIP efficiency, we probed in initial ChIP experiments for binding of PARP1 to the autoregulatory site in the *PARP1* promoter (Figure 5). A weak hairpin structure is stabilized by PARP1 binding [44], leading to transcriptional repression [45]. Therefore, if DNA-binding of PARP1 is compromised by automodification, this would be reflected by a reduced degree of PARP1 binding to the hairpin without suppression of PARylation. We immunoprecipitated PARP1 using the three different protocol variants (JLI/PJ34/BMB). Isolated DNA from input samples or recovered from immunoprecipitation was tested by semi-quantitative PCR for the presence of the promoter sequence. By comparing the PCR product signal intensities we detected a significant increase (1.7fold and 2.1fold for PJ34 and BMB, respectively) in ChIP efficiency if PARP activity was inhibited by PJ34 or by the more stringent fixation procedure. As control, we immunoprecipitated all samples with an irrelevant monoclonal antibody (12F10) and tested these in PCR. None of them yielded any detectable amplification products, thus demonstrating specificity of the precipitation. These experiments proved that PARylation affects protein binding to DNA at least in the case of PARP1. To further analyze the impact of PARylation on ChIP efficiency, we chose other transcription factors as well as histone H1 as a reported high-affinity binder to PAR [46], and tested their presence at published binding sites (Table S1) using the JLI and BMB protocols in parallel.

**Figure 6:**
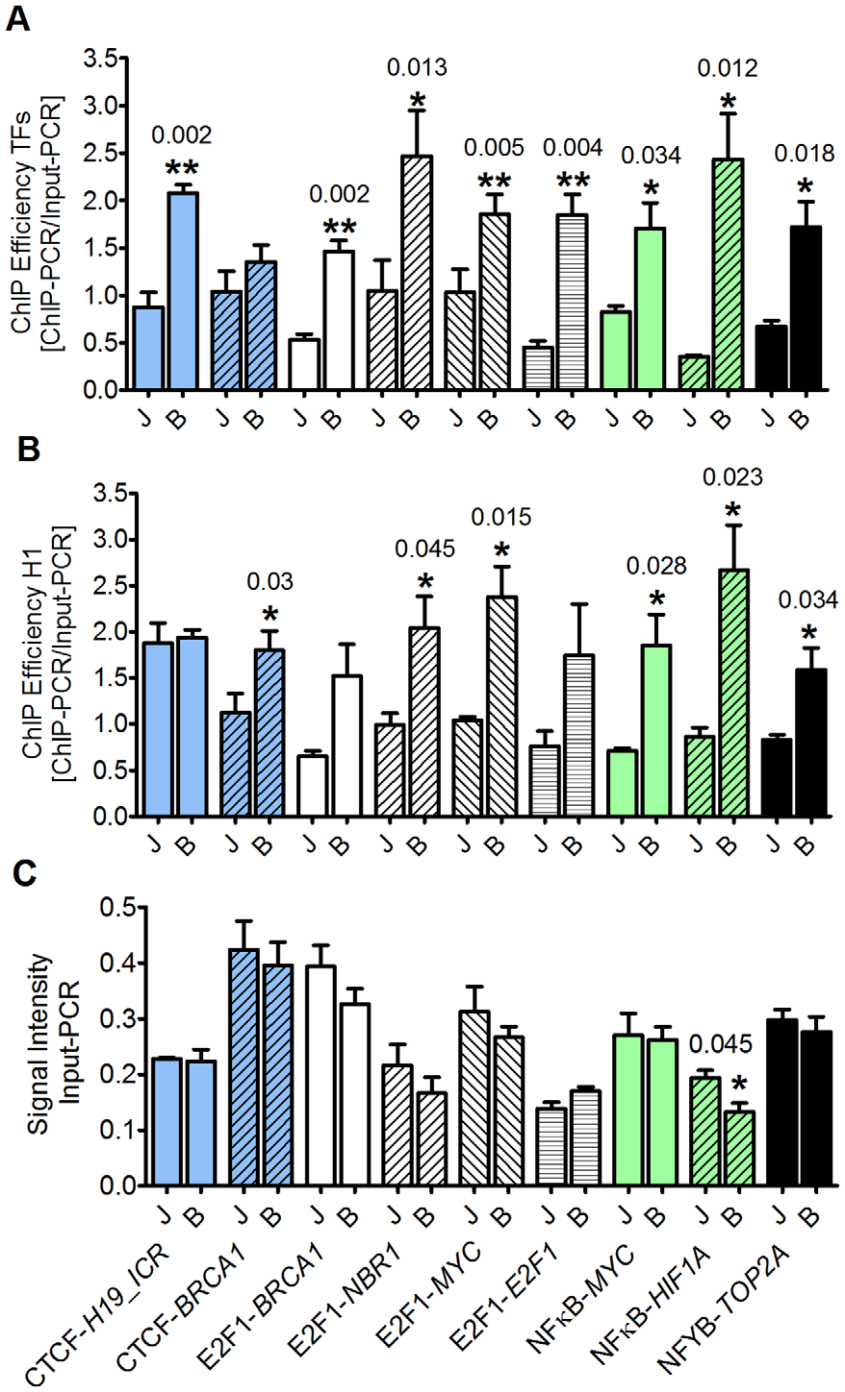
PARylation affects binding of transcription factors and histone H1 differently dependent on the binding site. Preparation of chromatin and ChIP was done by JLI and BMB protocol, respectively. Three independent chromatin preparations were analyzed by three independent PCRs each (panels A, C), or by only one PCR each (panel B) due to lack of material. (**A**) Suppression of PARylation improves ChIP efficiency in general, but with some specificity. Column color code: blue: ChIP with anti-CTCF antibody; white: ChIP with anti-E2F1 antibody; green: ChIP with anti-p65/RELA (NFκB) antibody; black: ChIP with anti-NFYB antibody. Respective binding sites and promoters are indicated on Y-axis of panel C. J = JLI protocol; B = BMB protocol. Error bars represent mean±s.e.m. (N = 3), exact P-values are indicated; data were analyzed by unpaired t-test. Note that CTCF binding is affected by PARylation at the *H19_ICR* locus, but not at the *BRCA1* promoter. (**B**) PARylation impacts on histone H1 binding independent of transcription factors. ChIP was performed with anti-H1 antibody and analyzed for binding at the same positions as in (A). Respective binding sites and promoters are indicated on Y-axis of panel C. Coding was maintained to simplify comparison. Error bars represent mean±s.e.m. (N = 3), exact P-values are indicated; data were analyzed by unpaired t-test. Note that H1 binding at the CTCF sites is oppositely affected by PARylation. (**C**) Increased formaldehyde concentration during fixation does not impact on PCR efficiency. Product signal intensities from input PCRs were compared. Respective binding sites and promoters are indicated on Y-axis. J = JLI protocol, B = BMB protocol. Coding was maintained to simplify comparison between panels. Only NFκB/RELA binding to *HIF1A* promoter displayed border-line significance in PCR efficiency. Error bars represent mean±s.e.m. (N = 3), *P = 0.045; data were analyzed by unpaired t-test.

Suppressing PARylation increased significantly ChIP efficiency in general, but affected specific promoters selectively (Figure 6A). Most prominently, ChIP efficiency was increased more than twofold at the *H19_ICR* imprinting control region, already known to be sensitive to the presence of PAR [47], but PARylation did not change binding of the transcription factor and insulator protein CTCF to the *BRCA1* promoter. Binding of E2F1 to sites in 4 different promoters was significantly increased, similar to binding of NFκB (subunit RELA) to *HIF1A* and *MYC* promoters and NFYB to the *TOP2A* promoter. Interestingly, comparing ChIP efficiencies between transcription factors and H1 at the same site showed in some cases an opposite behavior (compare Figure 6A and 6B). Prominent examples are again the CTCF binding sites: Whereas suppression of polymer synthesis increased occupancy of CTCF at the *H19_ICR* locus, H1 binding was not altered, and at the *BRCA1* promoter regulation was inverse, as PARylation affected significantly H1 binding, but not CTCF. In order to exclude any influence of formaldehyde concentration on efficiency of the subsequent PCR reaction, we analyzed input fragment intensities after PCR. In no case a significant change in PCR efficiency was detected except for the NFκB site in the *HIF1A* promoter (Figure 6C). To exclude chromatin alterations induced by prolonged incubation with PARP inhibitors and to control for possible changes due to increased formaldehyde concentrations at different sites, we performed additional experiments using two pairs of antibodies with respective promoter sequences, i.e. CTCF with *BRCA1* promoter or *H19-ICR*, and NFκB with *MYC* or *HIF1A* (see Materials and Methods S1 for experimental setup). In this set, PJ34 was added immediately before application of 1% formaldehyde (JLI protocol) instead of the overnight incubation and compared to chromatin fixed with standard JLI procedure in parallel. As summarized in Figure S2, short-term incubation with PJ34 led to similar results regarding PAR suppression and ChIP efficiency as fixation with 3.7% formaldehyde. These data support our findings that standard fixation procedures employing 1% formaldehyde (or even less) artificially induce poly(ADP-ribosyl)ation and chromatin alterations, which can be abrogated by suppressing PAR formation, either by PARP inhibition or fixation with 3.7% formaldehyde.

In summary, our data demonstrate that suppressing PAR production during ChIP fixation significantly impacts on the results obtained by this method. Therefore, we suggest in future ChIP experiments either the use of PARP inhibitors or 3.7% formaldehyde as fixative to avoid induction of PARylation and resulting artifacts.

## Discussion

**Figure 7:**
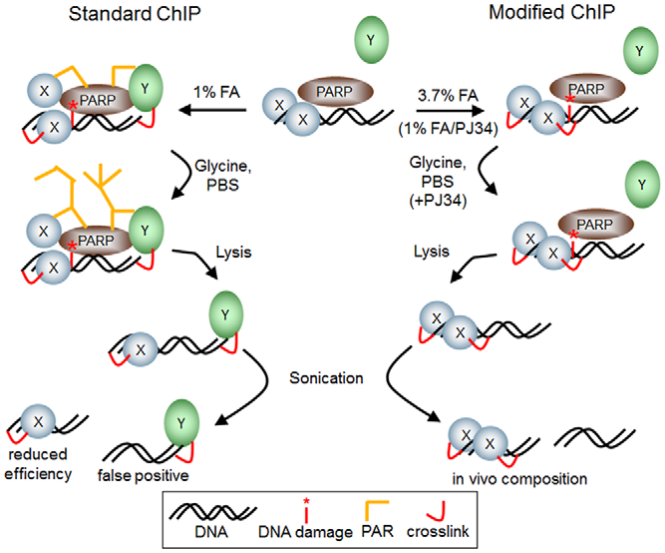
Model of PAR-dependent chromatin remodeling during ChIP fixation. On the left side, standard ChIP protocol leads to PARP (brown) activation and subsequent poly(ADP-ribose) formation (orange lines) by damaging DNA (red asterisk). This either dislodges proteins (blue, X) from DNA or attracts proteins (green, Y) to DNA with subsequent crosslinking (red arc). Therefore, after lysis and sonication, immunoprecipitated proteins can be present either in wrong amounts (reduced efficiency), or proteins are crosslinked that are not present on DNA in physiological conditions (false positive). On the right side, using 3.7% formaldehyde for 10 min as fixation protocol or treatment with the PARP inhibitor PJ34 throughout the experiment until lysis abolishes PAR formation completely. Thus, chromatin composition is unaltered and reflects in vivo situation.

PARP activity has been implicated in many processes within a cell, and most of them are connected to genomic maintenance and fidelity [3]. These require the remodulation of chromatin, i.e. during DNA repair, replication, telomere maintenance and transcription. Most recently, PARP1 has been shown to regulate specifically the expression of nuclear encoded genes involved in mitochondrial DNA repair [48]. PARPs have been shown to take part in all the above mentioned pathways, but most prominent is the enzymatic activity stimulated by DNA strand breaks. We show here that fixation of cells with low doses of formaldehyde also induces DNA damage signaling and PARylation (Figures 1, 2, 3) in human and mouse cell lines. The dose- and time-dependent decline in cellular PARylation during formaldehyde treatment relies most likely on successive inactivation of the enzyme by formaldehyde (Figure 1). Synthesis of poly(ADP-ribose) is mainly induced during the washing step after crosslinking in ChIP experiments (Figure 3). Using fibroblasts from different PARP knockout strains (Figure 2C), we determined PARP1 and PARP2 as independent sources of poly(ADP-ribose), but it could well be that also other putatively DNA-dependent PARPs like PARP3 [49] are activated by mild formaldehyde treatment. We could show that polymer formation is associated with a reduced ChIP-efficiency compared to two modified protocols in which we suppressed PAR synthesis. Testing for the autoregulatory site in the PARP1 promoter (Fig. 5), we achieved a twofold improvement of ChIP-efficiency by increasing formaldehyde concentration in the fixation step to 3.7%. Suppression of PARP activity by inhibitor treatment was slightly less effective (1.7fold), but still yielded significantly more product in semi-quantitative PCR than the original protocol (JLI). The reduced increase can be explained by the fact that PJ34 is a competitive inhibitor of NAD^+^ substrate binding, which may still lead to low and undetectable polymer synthesis, but nevertheless subtle changes in chromatin composition. Alternatively, as γH2AX formation is still observable after PJ34 application but abrogated by 3.7% formaldehyde fixation (Figure S1), this DNA damage marker may also lead to changes in the respective protein-DNA interactions, but only to a minor extent. Our results additionally show that both DNA damage markers, i.e. PAR and γH2AX, are induced by lesions of different quality, as there is little if any colocalization between them (Figure S1). Most likely, as PARPs are involved in regulation of base-excision repair, whereas γH2AX is a well-known marker of DNA double-strand breaks, PAR is synthesized at single-strand breaks and γH2AX foci appear at double-strand breaks. In summary, these data prove that chromatin composition is changed by the application of standard ChIP procedures per se. In order to test this in more detail we analyzed several transcription factors as well as histone H1 for presence at known binding sites. Our data revealed that - despite a general increase in ChIP efficiency - the effect of polymer on binding of proteins to DNA strongly depends on the specific site, and that the change in occupancy of the transcription factor or histone H1 can be inverse (Figure 6) as most evident in experiments utilizing the CTCF protein. Even only short pre-incubation with PJ34 had a very similar effect on ChIP efficiency (Figure S2). Thus, inhibition of PARylation leads not in all cases to increased ChIP efficiency, pointing to a specific regulation. PARP activity has been reported recently to rapidly recruit factors containing macro-domains like macroH2A [27] or Alc1 [29], and the PAR-dependent relocalization of XRCC1 to DNA strand breaks has long been known [35]. The attraction of proteins to sites of polymer production could lead to artificial crosslinking of these to DNA, yielding false positive results. On the other hand, dislodging proteins from DNA like histones or p53 [50] upon covalent modification would give no or not the correct amount of amplification product in PCR, thus underestimating the binding of a specific protein, which we have shown now for several important proteins used in ChIP applications. One might speculate that an important function of PARylation is the modulation of transcriptional activity at promoters after genotoxic stress in regard of fine-tuning transcription factor binding, as we mostly detected a mild two- to threefold enhancement in ChIP efficiency except for NFκB binding to the *HIF1A* promoter, which was increased nearly sevenfold. Figure 7 depicts our working model for PARP suppression in ChIP.

In summary, our results prove that standard ChIP protocols lead to artificial PAR production. As PAR changes chromatin organization by interaction with histones and transcription factors, this drastically influences the results of ChIP experiments. We show that inhibition of PARP either by PJ34 or by 3.7% formaldehyde is beneficial in maintaining the in vivo composition of chromatin, as both regimens prevent PARP activation. Our ChIP data reveal that this is in general associated with increased ChIP efficiency, and that comparing results obtained with the new and the old method can be utilized to uncover transcription factor binding sites that are especially sensitive or insensitive to poly(ADP-ribosyl)ation.

Deduced from our findings, previous data obtained by chromatin-immunoprecipitation have to be interpreted with caution, as they have been flawed by artificial PAR formation. In order to avoid non-physiological results caused by fixation artifacts by low formaldehyde concentration, increasing the formaldehyde concentration in the cross-linking step to 3.7% or including a PARP inhibitor is strongly recommended.

## Materials and Methods

### Ethics Statement

N/A

### Chemicals

Standard chemicals were purchased either from Sigma Aldrich (Munich, Germany) or from Merck (Darmstadt, Germany)

### Cell culture

HeLaS3 cells (DSMZ, Braunschweig, Germany) and mouse 3T3 fibroblast strains [51]–[53] were maintained in DMEM (Invitrogen, Darmstadt, Germany) with 10% fetal bovine serum (Biochrom, Berlin, Germany) and 1% penicillin/streptomycin (Invitrogen, Darmstadt, Germany) at 37°C/5% CO_2_. For long-term PARP inhibition, 2 µM PJ34 (Enzo Life Sciences, Lausen, Switzerland) was added to the cell culture medium at least 6 h in advance of subsequent processing.

### Immunostaining and fluorescence microscopy

Cells were seeded on glass cover slips and grown overnight. The next day, cells were treated with or without 1 mM H_2_O_2_ for 7 min at 37°C and fixed either with 100% methanol for 7 min at 4°C, with formaldehyde at varying concentrations and time periods as indicated, with formaldehyde and subsequent 100% methanol fixation to monitor PAR formation, or by standard ChIP protocols, respectively. After methanol fixation, cells were rinsed thrice with Tris-buffered saline (TBS) and stored in TBS for subsequent immunostaining. Formaldehyde fixation was stopped by addition of 0.1 M glycine/TBS at room temperature (RT) for 5 min, followed by 2x 3 min washing in TBS at RT. Cells were permeabilized with 0.4% Triton X-100 in TBS, followed by 2x 3 min washing in TBS at RT.

Cover slips were blocked in TBS/0.2% Tween20/1% BSA (TTB) at 30°C for 30 min, followed by incubation with primary antibody 10H [54] against poly(ADP-ribose) in blocking solution (1∶300). After 3 washes with TBS/0.2% Tween20 for 5 min at RT, secondary antibodies were applied, diluted 1∶500 in TTB for goat-anti-mouse Alexa546 and Alexa488, respectively (Molecular Probes/Invitrogen, Darmstadt, Germany) and incubated as above. Cover slips were washed again, incubated for 5 min at RT with 40 ng/ml 4′,6′-diamidino-2-phenylindole (DAPI, Sigma) and mounted on glass slides with AquaPolyMount (PolySciences, Eppelheim, Germany).

For γH2AX staining, cells were fixed with formaldehyde as above, followed by two washes in PBS and one in 50 mM NH_4_Cl in PBS for 10 min each. Cells were permeabilized with 0.2% TritonX-100 in PBS for 4 min at room temperature. After extensive washings with PBS, coverslips were incubated with 1% BSA in PBS for 30 min and then with anti-phosphoH2AX (Ser139, Millipore, Schwalbach, Germany) antibody diluted 1∶500 in PBS containing 10% normal goat serum for 1 h at RT. Alexa Fluor 546-conjugated goat anti-mouse antibody was used as secondary antibodies at a dilution of 1∶400. γH2AX-positive foci were visualized by laser scanning confocal microscopy with identical settings between experiments, and average intensity projections of 26 z planes of at least 100 nuclei/sample were measured. Standard immunofluorescence analyses were performed on a Zeiss Axiovert 200M mounted with 40x Plan-Neofluar (1.3NA) oil objective. Pictures were taken with Zeiss AxioCam MRm and Zeiss AxioVision4.5 software (Carl Zeiss Company, Göttingen, Germany). For confocal microscopy a Zeiss Laser Scanning Microscope LSM 510 Meta with 63x Plan-Apochromat (1.4NA) oil objective, Zeiss AxioCam MRm and Zen software was used. Samples were analyzed in 0.32 µm sections. Subsequent data processing was done with ImageJ 1.42q (MacBiophotonics) and Adobe Photoshop.

### Protocols for chromatin-immunoprecipitation **(**ChIP**)** and respective immunofluorescences

#### JLI [42]

10% crosslinking mix (11% formaldehyde/100 mM NaCl/ 0.5 mM EGTA/50 mM HEPES, pH8.0) was added to medium. After 10 min at room temperature, 10% 1.25 M glycine was added. After removing of the solution, plates were washed 2x with PBS (3 min each).

For immunofluorescence, PBS was aspirated and replaced by 0.4% TritonX100 in PBS for 5 min at room temperature for permeabilization. Cover slips were again washed 2x with PBS and blocked afterwards with blocking solution, followed by immunostaining procedure.

To yield chromatin suspension, cells were scraped in 1 ml lysis buffer (1% SDS/10mM EDTA, pH 8.0/50mM Tris-HCl, pH 8.0/1x complete protease inhibitor mix, Roche) after incubation for 5 min at 4°C. Suspensions were sonicated on ice for 10 sec at 50% output, with 2 min refractory period (Bandelin Sonoplus HD-070, tip MS73; Bandelin, Berlin, Germany). Concentration was adjusted to 1 µg/µl with lysis buffer and stored at 4°C.

For ChIP, suspensions were brought to room temperature and 420 µl were spun down for 5 min at maximum speed. 400 µl supernatant was mixed with 3.6 ml dilution buffer (1% Triton X-100/150mM NaCl/2mM EDTA, pH 8.0/20mM Tris-HCl, pH 8.0/1x complete protease inhibitor mix, Roche) and incubated with by distributor recommended amount of antibody (if no information available: 1 µg/ml final concentration). Samples were rotated over night at 4°C. 100 µl of on agarose-beads immobilized Protein G (ThermoScientific, Rockford/IL, USA) were resuspended in 1 ml 9∶1 dilution buffer:lysis buffer mix (DB∶LB) and pre-absorbed with 100 µg/ml BSA and 500 µg/ml sheared salmon sperm DNA overnight at 4°C on a rotator. On the next day, beads were washed twice with DB∶LB and resuspended in 1 ml DB∶LB. 100 µl of the beads suspension was added to each cell lysate and incubated for at least 2 h at 4°C on a rotator. Suspensions were spun down at 1000 *g* and supernatant was aspirated. Beads were washed 3x in 1 ml wash buffer (1% Triton X-100/0.1% SDS/150 mM NaCl/2 mM EDTA, pH 8.0/20 mM Tris-HCl, pH 8.0/1x complete protease inhibitor mix; Roche, Grenzach, Germany) and centrifuged as above. Beads were washed 1x in 1 ml final wash (1% Triton X-100/0.1% SDS/500 mM NaCl/2 mM EDTA, pH 8.0/20 mM Tris-HCl, pH 8.0/1x complete protease inhibitor mix, Roche) and immune-complexes were eluted by addition of 450 µl elution buffer (1% SDS/100 mM NaHCO_3_) 5 min at room temperature. 500 µg/ml ProteinaseK and RNaseA were added to each sample and incubated for additional 30 min at 37°C. Cross-linking was reversed by adding NaCl to a final concentration of 200 mM and incubation overnight at 65°C. Suspension was spun down and DNA from the supernatant was isolated by Phenol/Chloroform procedure and subsequent ethanol precipitation.

#### MLI [41]

****For immunofluorescence, cells on cover slips were washed once with PBS and solution was replaced with 1% formaldehyde in PBS. Cells were incubated at room temperature for 10 min and reaction was stopped by adding 1.25 M glycine to a final concentration of 0.11 M. Cells were washed 2x with ice cold PBS and permeabilized by 5 min in 0.4% TritonX100 in PBS at room temperature. Cover slips were again washed 2x with PBS and blocked afterwards with blocking solution, followed by immunostaining procedure.

#### UMC [43]

Cells on cover slips were fixed by adding formaldehyde directly to the growth medium to a final concentration of 1% and incubation for 10 min at 37°C. Reaction was stopped by adding glycine to a final concentration of 125 mM and incubation for 5 min at 37°C. Cover slips were rinsed twice with ice-cold 1x PBS/0.5 mM EDTA and incubated for 5 min at room temperature with 0.4% TritonX100 to permeabilize cells. Cover slips were again washed 2x with PBS and blocked afterwards with blocking solution, followed by immunostaining procedure.

### Chromatin immunoprecipitation protocols variations

Protocols from three different public available sources were used in this study. See Materials and Methods for detailed protocols: JLI [42]; MLI [41]; UMC [43].

#### Variations of JLI protocol

PJ34: 2 µM of PARP inhibitor PJ34 was included in all protocol steps until lysis.

BMB: Cells were incubated for 10 min with 3.7% formaldehyde instead of 1% as in JLI. Therefore, number of sonication cycles had to be increased two- to fourfold in order to achieve similar fragment sizes of genomic DNA.

Antibodies were purchased from Active Motif (CTCF, E2F1; Carlsbad/CA, USA) and Santa Cruz Biotechnologies (H1, NFκB p65/RELA, NFYB; Heidelberg, Germany). CII10 and 12F10 were kind gifts of G.G. Poirier (Quebec/Canada) and W. Bodemer (Göttingen/Germany), respectively.

### Polymerase chain reaction after chromatin-immunoprecipitation

#### PARP1 promoter

25 ng of input DNA was amplified in comparison to 1 µl of ChIP-sample by PCR with KOD HotStart polymerase according to manufacturer's instructions (Novagen/Merck, Darmstadt, Germany). PCR was performed in 33 cycles (20 s 95°C/10 s 59°C/5 s 70°C) and products were resolved on 5% polyacrylamide gels.

#### Other promoters

For input, lysed material was directly subjected to PCR amplification. ChIP amplification was performed similar as above in 35 cycles (20 s 95°C/10 s annealing temperature/5 s 70°C). Fragments were resolved by 2.5% agarose gel electrophoresis.

Primer sequences and respective annealing temperatures are listed in Table S1.

### Evaluation of ChIP efficiency by PCR

Three independent ChIP experiments were analyzed by three subsequent PCRs each and averages were compared (N = 3). For H1, each ChIP was followed by only one subsequent PCR due to lack of material. Fragment signal intensities of input and ChIP PCRs were analyzed by Fuji-LAS1000 and Aida3.1 software (Fuji, Düsseldorf, Germany).

### Statistical evaluation

Samples were analyzed with GraphPad software Prism5 or Instat3 (GraphPad, La Jolla/CA, USA). Statistical tests used are indicated in the respective figure legends. A P-value <0.05 was considered significant, and exact P-values are reported if possible.

## Supporting Information

Figure S1 (**A**) PAR and γH2AX staining in HeLa cells and human VH7 fibroblasts after JLI and BMB fixation. Human tumor (HeLa S3) and normal (VH7 fibroblast) cells were fixed by JLI (1% FA) or BMB (3.7% FA) protocol and analyzed for appearance of PAR and γH2AX foci. Whereas 1% FA clearly induces PAR as well as γH2AX, both DNA damage markers are abolished (VH7) or greatly diminished (HeLa S3) if 3.7% FA is used for fixation. (**B**) PAR and γH2AX staining in HeLa cells and human VH7 fibroblasts after JLI and JLI+PJ34 fixation. Human cells were fixed by JLI (1% FA) or JLI+PJ34 protocol and analyzed for appearance of PAR and γH2AX foci. Whereas 1% FA clearly induces both PAR and γH2AX, simultaneous PJ34 application abolishes PAR synthesis in tumor (HeLa) and normal (VH7) cells, but has no impact on suppressing γH2AX formation. (**C**) PAR and γH2AX are markers of DNA lesions of different quality. HeLa cells were fixed by JLI protocol and immunostained for PAR (green) and γH2AX (red). Cells were analyzed for colocalization of PAR and γH2AX foci using ImageJ software. As evident from the respective Pearson's analysis (data in lower panel), foci show some, but only low degree of colocalization of both DNA damage markers after JLI fixation. As there is no PAR synthesis after PJ34 treatment, there is also no colocalization detectable. For better visualization, fluorescence intensities along a track line were analyzed. (**D**) PAR and γH2AX are markers of DNA lesions of different quality. VH7 cells were fixed by JLI protocol and immunostained for PAR (green) and γH2AX (red). Cells were analyzed for colocalization of PAR and γH2AX foci using ImageJ software. In the upper panel the image of a segmented nucleus and the corresponding scatter plot and correlation coefficients are shown. As evident from Pearson's analysis, foci show no colocalization of both DNA damage markers after JLI fixation. For better visualization of foci distribution, the lower panel displays the fluorescence intensities of green and red along the indicated track line. As there is no PAR synthesis after PJ34 treatment or 3.7% formaldehyde, colocalization analysis was not performed.
(PDF)

Figure S2 **Application of PJ34 immediately before JLI fixation is similar to fixation with BMB protocol in ChIP efficiency.** HeLa cells were fixed by JLI protocol with 10 µM PJ34 application immediately before fixation. Chromatin was immunoprecipitated as indicated by CTCF or NFκB antibody as described. Precipitates were analyzed by PCR for the same DNA sequences as tested in Figure 6. Three independent chromatin preparations for each fixation were analyzed in parallel by one IP followed by one PCR. Intensity from an unspecific ChIP-PCR was subtracted from specific ChIP-PCR intensity and results were normalized to input-PCR intensity. Data from respective samples analyzed in parallel were subjected to two-tailed paired t-test. Actual P-values are indicated. Figure S2A shows results for CTCF antibody ChIP at *BRCA1* promoter and *H19_ICR* region, Figure S2B for NFκB antibody ChIP at *MYC* and *HIF1A* promoters.
(TIF)

Materials and Methods S1 **Immunofluorescence analysis and modified ChIP procedure for experiments described in** **Supporting Information.** Colocalization analysis between PAR and γH2AX was performed according to [55].
(DOC)

Table S1 **Promoters and regions tested by ChIP for binding of denoted proteins, amplicon position and primer sequences**.
(DOC)
