# Supplementary material for: Chromatin Composition Is Changed by Poly(ADP-ribosyl)ation during Chromatin Immunoprecipitation
Source: PLoS One. 2012 Mar 30;7(3):e32914. doi: 10.1371/journal.pone.0032914 (PMC3316553; doi:10.1371/journal.pone.0032914)

Figure S1A:
PAR and γH2AX staining in HeLa cells and human VH7 fibroblasts after JLI and BMB fixation

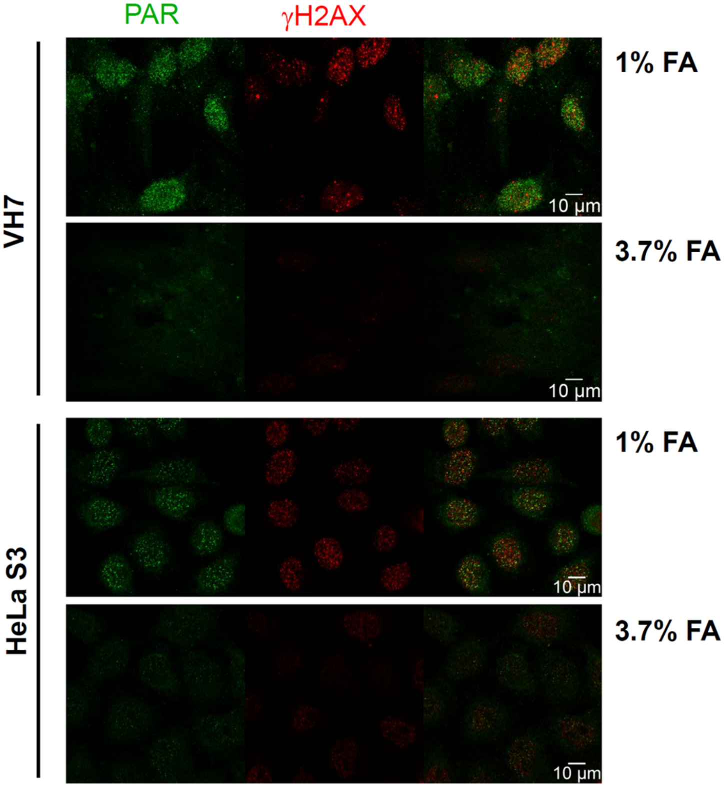

Figure S1B:
PAR and γH2AX staining in HeLa cells and human VH7 fibroblasts after JLI and JLI+PJ34 fixation

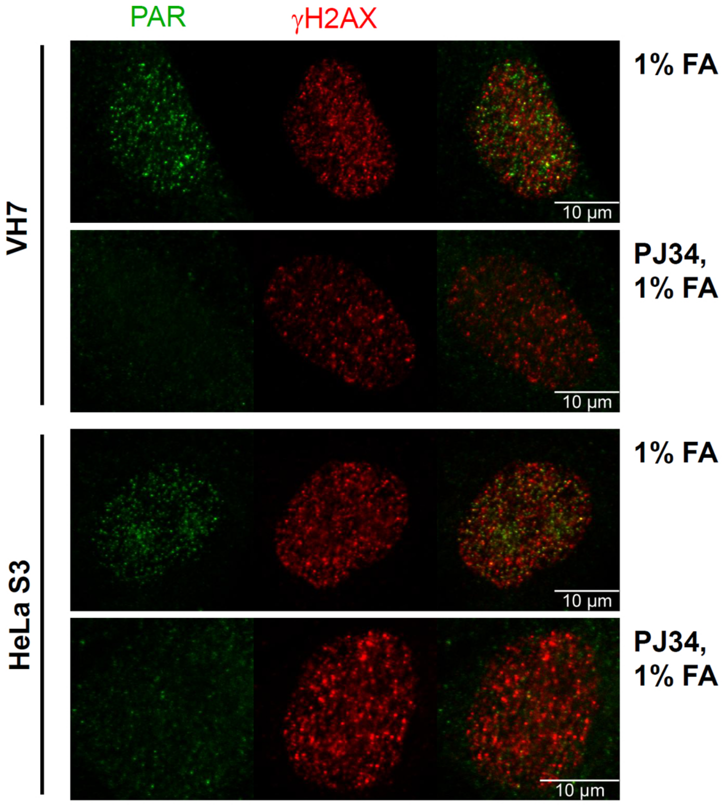

Figure S1C: PAR and γH2AX are markers of DNA lesions of different quality

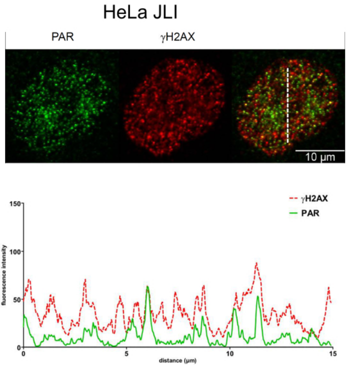

ImageJ – PSC colocalization
Segmentation: raw data – whole nucleus

Pixels 137662
Pearson's r = 0.04506745861002938
Spearman's r = 0.01649989621407877
Spearman's r (Ties) = 0.01508194732038942

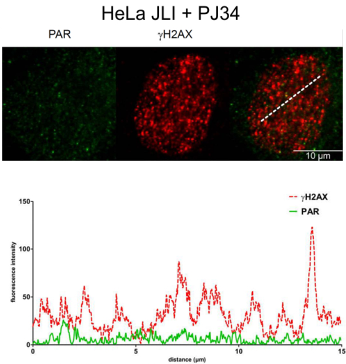

ImageJ – PSC colocalization
Segmentation: raw data – whole nucleus

Pixels 203112
Pearson's r = -0.05933110245850771
Spearman's r = -0.018300500631740935
Spearman's r (Ties) = -0.032582525027772063

Figure S1D: PAR and γH2AX are markers of DNA lesions of different quality

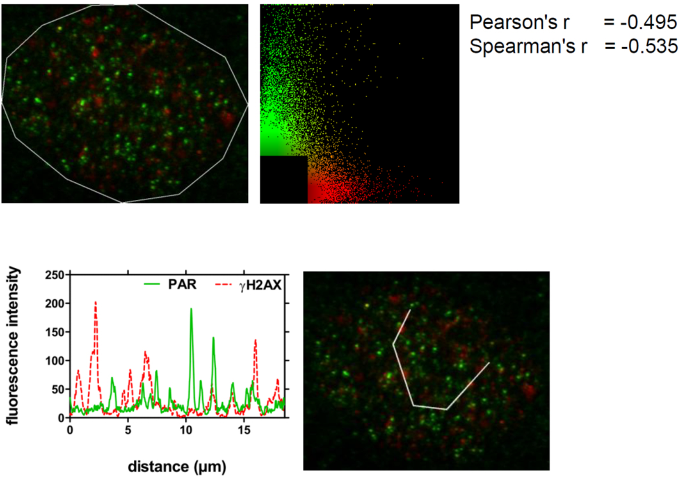

Supplement: Figure S1 — (A) PAR and γH2AX staining in HeLa cells and human VH7 fibroblasts after JLI and BMB fixation. Human tumor (HeLa S3) and normal (VH7 fibroblast) cells were fixed by JLI (1% FA) or BMB (3.7% FA) protocol and analyzed for appearance of PAR and γH2AX foci. Whereas 1% FA clearly induces PAR as well as γH2AX, both DNA damage markers are abolished (VH7) or greatly diminished (HeLa S3) if 3.7% FA is used for fixation. (B) PAR and γH2AX staining in HeLa cells and human VH7 fibroblasts after JLI and JLI+PJ34 fixation. Human cells were fixed by JLI (1% FA) or JLI+PJ34 protocol and analyzed for appearance of PAR and γH2AX foci. Whereas 1% FA clearly induces both PAR and γH2AX, simultaneous PJ34 application abolishes PAR synthesis in tumor (HeLa) and normal (VH7) cells, but has no impact on suppressing γH2AX formation. (C) PAR and γH2AX are markers of DNA lesions of different quality. HeLa cells were fixed by JLI protocol and immunostained for PAR (green) and γH2AX (red). Cells were analyzed for colocalization of PAR and γH2AX foci using ImageJ software. As evident from the respective Pearson's analysis (data in lower panel), foci show some, but only low degree of colocalization of both DNA damage markers after JLI fixation. As there is no PAR synthesis after PJ34 treatment, there is also no colocalization detectable. For better visualization, fluorescence intensities along a track line were analyzed. (D) PAR and γH2AX are markers of DNA lesions of different quality. VH7 cells were fixed by JLI protocol and immunostained for PAR (green) and γH2AX (red). Cells were analyzed for colocalization of PAR and γH2AX foci using ImageJ software. In the upper panel the image of a segmented nucleus and the corresponding scatter plot and correlation coefficients are shown. As evident from Pearson's analysis, foci show no colocalization of both DNA damage markers after JLI fixation. For better visualization of foci distribution, the lower panel displays the fluorescence intensities of green [file pone.0032914.s001.pdf]
