# Supplementary figures and images for: Chromatin Composition Is Changed by Poly(ADP-ribosyl)ation during Chromatin Immunoprecipitation
Source: PLoS One. 2012 Mar 30;7(3):e32914. doi: 10.1371/journal.pone.0032914 (PMC3316553; doi:10.1371/journal.pone.0032914)

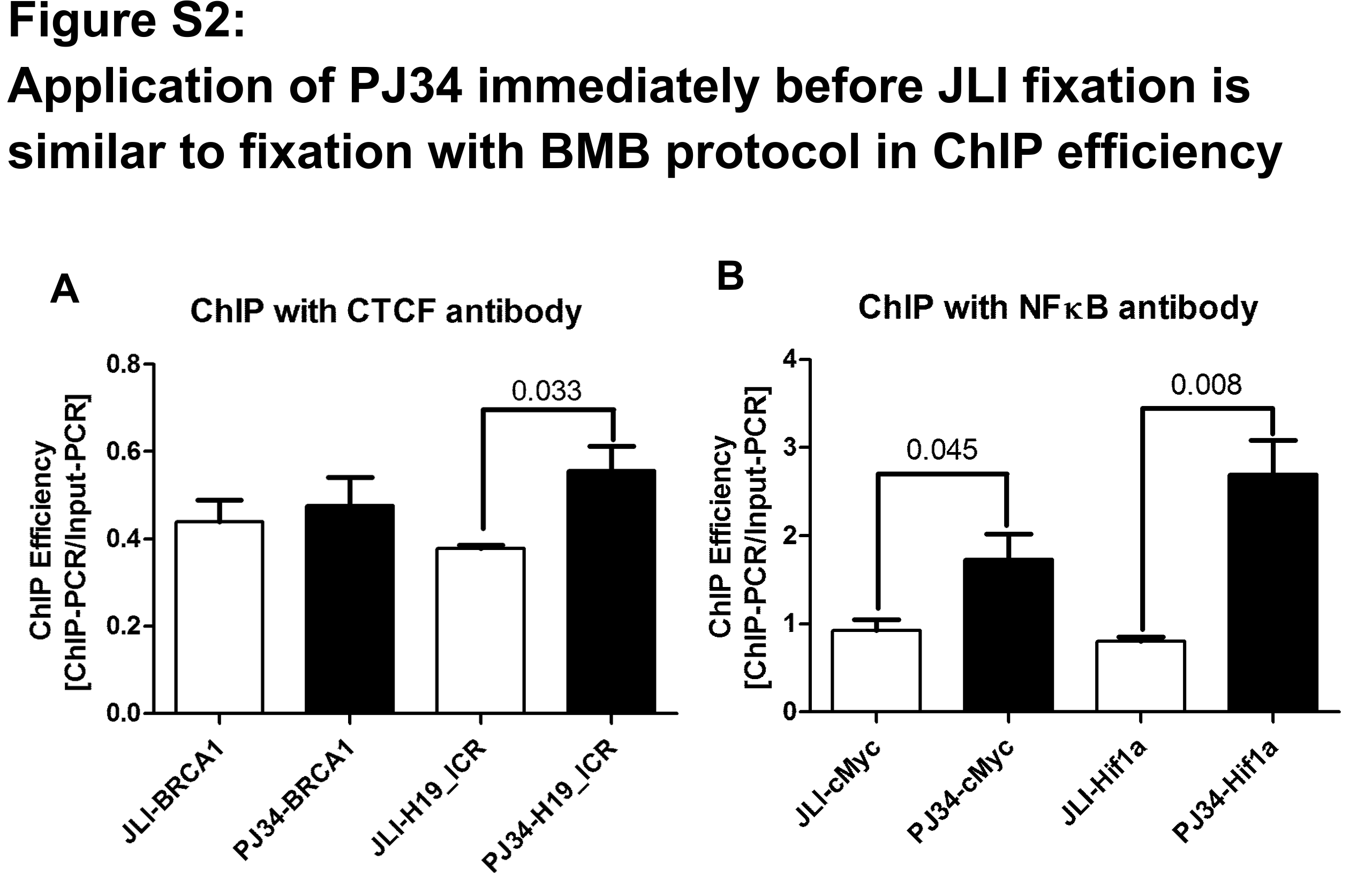

Supplement: Figure S2 — Application of PJ34 immediately before JLI fixation is similar to fixation with BMB protocol in ChIP efficiency. HeLa cells were fixed by JLI protocol with 10 µM PJ34 application immediately before fixation. Chromatin was immunoprecipitated as indicated by CTCF or NFκB antibody as described. Precipitates were analyzed by PCR for the same DNA sequences as tested in Figure 6. Three independent chromatin preparations for each fixation were analyzed in parallel by one IP followed by one PCR. Intensity from an unspecific ChIP-PCR was subtracted from specific ChIP-PCR intensity and results were normalized to input-PCR intensity. Data from respective samples analyzed in parallel were subjected to two-tailed paired t-test. Actual P-values are indicated. Figure S2A shows results for CTCF antibody ChIP at BRCA1 promoter and H19_ICR region, Figure S2B for NFκB antibody ChIP at MYC and HIF1A promoters. (TIF) [file pone.0032914.s002.tif]
