# Supplementary material for: Chromatin Composition Is Changed by Poly(ADP-ribosyl)ation during Chromatin Immunoprecipitation
Source: PLoS One. 2012 Mar 30;7(3):e32914. doi: 10.1371/journal.pone.0032914 (PMC3316553; doi:10.1371/journal.pone.0032914)
Supplement: Materials and Methods S1 — Immunofluorescence analysis and modified ChIP procedure for experiments described in Supporting Information. Colocalization analysis between PAR and γH2AX was performed according to [55]. (DOC) [file pone.0032914.s003.doc]

**SUPPORTING INFORMATION**

**Materials and Methods**

Cell culture

HeLaS3 human tumor cells and VH7 normal human fibroblasts (DSMZ, Braunschweig, Germany) were maintained in DMEM (Invitrogen, Darmstadt, Germany) with 10% fetal bovine serum (Biochrom, Berlin, Germany) and 1% penicillin/streptomycin (Invitrogen, Darmstadt, Germany) in a humidified atmosphere incubator at 37°C/5% CO_2_/air. For immunofluorescence, cells were seeded on 1 cm diameter glass cover slips on 24-well plates and grown for at least 36 h.

Fixation and immunofluorescence

Cells were fixed by standard JLI protocol or by BMB protocol in parallel as described in the manuscript Materials and Methods. Immediately before addition of the crosslinking mix, 10 µM PJ34 was applied to yield respective PJ34 cultures (short term treatment). Incubation with PJ34 was not longer than 5 min at room temperature. After fixation and quenching the reaction by addition of 10% volume of 1.25 M glycine, solutions were replaced by PBS with or without 10 µM PJ34, respectively. This washing was repeated once. To suppress background fluorescence signals, cells were fixed afterwards with methanol (-20°C) for 7 min at 4°C, followed by 2 washes for 3 min at room temperature with PBS. Immunostaining procedure was as described as in Materials and Methods in the manuscript. Antibodies used were 10H (1:300) in combination with Goat-anti-Mouse Alexa488 (Sigma Aldrich, Buchs, Switzerland; 1:400) as well as anti-γH2AX (Novus Biologicals, Cambridge, GB, rabbit polyclonal; 1:200) and Goat-anti-Rabbit Alexa594 (Sigma Aldrich, Buchs, Switzerland; 1:400) in TTB.

Confocal microscopy

For colocalization analysis 0.8 μm sections were recorded using a Zeiss Laser Scanning Microscope LSM 700 with 63x Plan-Apochromat (1.4NA) objective (Carl Zeiss Company, Göttingen, Germany). Colocalization analysis was performed using ImageJ and the PSC colocalization plugin [[55]](#_ENREF_1). Line intensity plots were generated after applying a median filter (radius 1 pixel) to both image channels.

Fixation, fragmentation and chromatin immunoprecipitation

Two 15-cm dishes for each condition were used. PJ34 cultures were incubated no longer than 5 min with 10 µM PJ34 before addition of crosslinking mix, following JLI protocol. Subsequent steps until lysing cells contained also 10 µM PJ34. JLI cultures were treated as described in the manuscript. Chromatin fragmentation and immunoprecipitation was performed as described in the manuscript Materials and Methods. Antibodies used were anti-CTCF (Active Motif) and NFκB (Santa Cruz Biotechnologies). Eluates from ChIP and DNA from input chromatin were purified by phenol/chloroform and subsequent ethanol precipitation.

Polymerase chain reaction

Purified input DNA was diluted 1:10 and subjected to PCR amplification in parallel to ChIP purified DNA. DNA volume was 5% of total PCR volume and reaction was performed with KOD HotStart polymerase according to manufacturer’s instructions (Novagen/Merck, Darmstadt, Germany). PCR was performed in 35 cycles (20 s 95°C/10 s annealing temperature/5 s 70°C) starting with 2 min activation at 95°C. Fragments were resolved by 2.5% agarose gel electrophoresis. Primer sequences and respective annealing temperatures are listed in Supporting Table 1.
