# Supplementary material for: Chromatin Composition Is Changed by Poly(ADP-ribosyl)ation during Chromatin Immunoprecipitation
Source: PLoS One. 2012 Mar 30;7(3):e32914. doi: 10.1371/journal.pone.0032914 (PMC3316553; doi:10.1371/journal.pone.0032914)
Supplement: Table S1 — Promoters and regions tested by ChIP for binding of denoted proteins, amplicon position and primer sequences. (DOC) [file pone.0032914.s004.doc]

**Table S1: Promoters and regions tested by ChIP for binding of denoted proteins, amplicon position and primer sequences**

| Protein | Promoter | primer sequence fwd / rev (5’-3’); annealing temperature [°C] | amplicon position relative to ATG |
| --- | --- | --- | --- |
| PARP1 | *PARP1* | TGTCAACCCAGAGATGGCAT / AACTACTCGGGAGGCTGAA; 59 | -1693 to –1931 |
| CTCF | *BRCA1* | CTGCTTCCTTACCAGCTTCC / AGGGAGACTACAATTCCCATCC; 61 | -2367 to –1954 |
| CTCF | *H19_ICR* | CCTTCGGTCTCACCGCCTG / CCTTAGACGGAGTCGGAGCTG; 69 | (-51029 to –50673) |
| E2F1 | *BRCA1* | CGAGAGACGCTTGGCTCTTTCTGT / GCCCAGTTATCTGAGAAACCCCAC; 61 | -1429 to –1216 |
| E2F1 | *MYC* | GCTTCTCAGAGGCTTGGCG / CGAAAAAAATCCAGCGTCTAAGC; 61 | +392 to +516 |
| E2F1 | *E2F1* | AGGAACCGCCGCCGTTGTTCCCG / GCTGCCTGCAAAGTCCCGGCCAC; 69 | -229 to –106 |
| E2F1 | *NBR1* (this work) | CGAGAGACGCTTGGCTCTTTCTGT / GCCCAGTTATCTGAGAAACCCCAC; 61 | -5500 to –6004 |
| NFκB/RELA | *MYC* | ACTTTGCACTGGAACTTACAACAC / CGAAAAAAATCCAGCGTCTAAGC; 61 | +383 to +516 |
| NFκB/RELA | *HIF1A* | GAACAGAGAGCCCAGCAGAG / CTGAGGTGGAGGCGGGTTC; 69 | -536 to –137 |
| NFYB | *TOP2A* | GGTGCCTTTTGAAGCCTCTCTAG / GCTCCACTTGAACCTTCCTTTAGC; 61 | -306 to-112 |

1. Soldatenkov VA, Chasovskikh S, Potaman VN, Trofimova I, Smulson ME, et al. (2002) Transcriptional repression by binding of poly(ADP-ribose) polymerase to promoter sequences. J Biol Chem 277: 665-670.

2. Xu J, Huo D, Chen Y, Nwachukwu C, Collins C, et al. (2009) CpG island methylation affects accessibility of the proximal BRCA1 promoter to transcription factors. Breast Cancer Res Treat 120: 593-601.

3. Yu W, Ginjala V, Pant V, Chernukhin I, Whitehead J, et al. (2004) Poly(ADP-ribosyl)ation regulates CTCF-dependent chromatin insulation. Nat Genet 36: 1105-1110.

4. Watanabe S, Ishida S, Koike K, Arai K (1995) Characterization of cis-regulatory elements of the c-myc promoter responding to human GM-CSF or mouse interleukin 3 in mouse proB cell line BA/F3 cells expressing the human GM-CSF receptor. Mol Biol Cell 6: 627-636.

5. Leung JY, Ehmann GL, Giangrande PH, Nevins JR (2008) A role for Myc in facilitating transcription activation by E2F1. Oncogene 27: 4172-4179.

6. Barre B, Perkins ND (2007) A cell cycle regulatory network controlling NF-kappaB subunit activity and function. Embo J 26: 4841-4855.

7. Bonello S, Zahringer C, BelAiba RS, Djordjevic T, Hess J, et al. (2007) Reactive oxygen species activate the HIF-1alpha promoter via a functional NFkappaB site. Arterioscler Thromb Vasc Biol 27: 755-761.

8. van Uden P, Kenneth NS, Rocha S (2008) Regulation of hypoxia-inducible factor-1alpha by NF-kappaB. Biochem J 412: 477-484.

9. Stros M, Polanska E, Struncova S, Pospisilova S (2009) HMGB1 and HMGB2 proteins up-regulate cellular expression of human topoisomerase IIalpha. Nucleic Acids Res 37: 2070-2086.
